# Are type III–IV muscle afferents required for a normal steady-state exercise hyperpnoea in humans?

**DOI:** 10.1113/jphysiol.2013.261925

**Published:** 2013-09-30

**Authors:** Jerome A Dempsey, Grégory M Blain, Markus Amann

**Affiliations:** 1 John Rankin Laboratory of Pulmonary Medicine, University of Wisconsin – MadisonMadison, WI, USA; 2 Faculty of Sports Sciences, University of Nice Sophia AntipolisNice, France; 3 Department of Internal Medicine, University of UtahSalt Lake City, UT, USA

## Abstract

When tested in isolation, stimuli associated with respiratory CO_2_ exchange, feedforward central command and type III–IV muscle afferent feedback have each been shown to be capable of eliciting exercise-like cardio-ventilatory responses, but their relative contributions in a setting of physiological exercise remains controversial. We reasoned that in order to determine whether any of these regulators are *obligatory* to the exercise hyperpnoea each needs to be removed or significantly diminished in a setting of physiological steady-state exercise, during which all recognized stimuli (and other potential modulators) are normally operative. In the past few years we and others have used intrathecal fentanyl, a μ-opiate receptor agonist, in humans to reduce the input from type III–IV opiate-sensitive muscle afferents. During various types of intensities and durations of exercise a sustained hypoventilation, as well as reduced systemic pressure and cardioacceleration, were consistently observed with this blockade. These data provide the basis for the hypothesis that type III–IV muscle afferents are obligatory to the hyperpnoea of mild to moderate intensity rhythmic, large muscle, steady-state exercise. We discuss the limitations of these studies, the reasons for their disagreement with previous negative findings, the nature of the muscle afferent feedback stimulus and the need for future investigations.

## Introduction

The ventilatory response to rhythmic exercise is an exquisite example of a highly efficient homeostatic response. Not only does alveolar ventilation rise in precise proportion to respiratory CO_2_ exchange, but the nature of each hyperpnoeic breath is also tightly controlled in terms of increasing frequency *vs*. tidal volume, breath duty cycle and recruitment of inspiratory and expiratory musculature of the chest and abdominal walls as well as the upper airway dilator musculature. These responses are dedicated to both regulate the arterial blood gases and acid–base status as well as to minimize the work, and the metabolic and circulatory costs, required to produce up to 10- to 20-fold increases in ventilation. Furthermore, as eloquently espoused by the late Brian Whipp (1937–2011), in health with increasing exercise intensity, anatomical (airway) dead space volume (*V*_d_) increases as a linear function of increasing tidal volume (*V*_T_) and *V*_d_/*V*_T_ falls as a hyperbolic function of increasing *V*_T_. Thus, overall expiratory minute ventilation (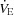 )/rate of CO_2_ production (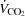 ) is reduced proportionately with increasing exercise intensities so as to maintain constant the ratio of alveolar ventilation to CO_2_ production 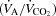 and thus maintain normocapnia over the range of mild to moderate intensity exercise in the steady state (Whipp, 1975):


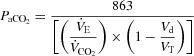
(1)

where 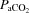 is the arterial partial pressure of CO_2_.

Of relevance to this mysterious capability of the control system to somehow ‘know’ to increase total ventilation only to levels commensurate with the demands for pulmonary CO_2_ exchange, is the observation that with healthy ageing, reduced lung elastic recoil increases *V*_d_/*V*_T_ at rest and during exercise. Despite this age-dependent disruption to gas exchange efficiency, 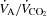 and normocapnia are maintained during exercise in the elderly, similarly to that in the young adult, as 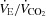 is adjusted further upward in the elderly to accommodate their higher *V*_d_/*V*_T_ (Johnson *et al*. 1969; Forster *et al*. 1924).

In our brief review we first outline what we and others consider to be three key mechanisms which probably contribute significantly to the exercise hyperpnoea and then focus specifically on recent evidence in humans supporting an essential role for type III–IV muscle afferents to the steady-state hyperpnoea.

## Three key mechanisms underlie exercise hyperpnoea

A century plus long debate has centred upon the tight link of 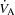 to 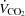, i.e. what is the nature of the mechanism(s) underlying this link? Three candidates have proven worthy based on their capability to drive ventilation – at least when studied *in isolation* – namely CO_2_ exchange at the lung, central command in proportion to locomotor muscle recruitment (feed-forward) and muscle afferent feedback.

### Pulmonary CO_2_ exchange

If arterial 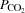 is indeed a key regulated variable then ‘CO_2_’ should have a major voice in its own regulation. In addition to the apparently inevitable proportional link of 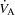 to 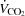 outlined above there is ample experimental evidence to prove cause (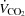 ) and effect (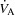 ) (Whipp, 1975): (a) increasing or decreasing CO_2_ flow to the lung, by itself, using extracorporeal circulation in a resting animal elicits a near isocapnic hyperpnoea or hypopnoea – at least over relatively small changes in 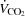 near eupnoea (Yamamoto & Edwards, 2008; Phillipson *et al*. 1992,1990; Green & Sheldon, 1995); (b) increasing 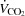 by increasing the respiratory quotient via carbohydrate ingestion elicits an isocapnic hyperpnoea in humans at rest (Douglas & Priestley, 1966; Whipp, 1975); (c) with sinusoidal variation in work rate over different durations, the ventilatory response varies more closely with 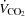 rather than work rate (Casaburi *et al*. 1984); and (d) electrical stimulation of limb muscle contractions to induce increases in 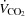 elicits an isocapnic hyperpnoea and this persists following spinal cord lesioning (in most studies) in humans and animals – again over a limited range of 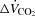 (Green & Sheldon, 1995; Adams *et al*. 1984). Further, it follows that this 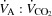 link probably also underlies the near-identical resting 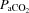 commonly observed among humans who vary in body mass by as much as 150 kg with a threefold variation in 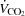 (Dempsey *et al*. 2012).

Despite these compelling arguments it is still not entirely clear how and where a signal that is proportional to pulmonary CO_2_ exchange is sensed. The carotid chemoreceptors provide an important tonic contribution to the eupnoeic drive to breathe at rest (Blain *et al*. 1988), but based on chemo-denervation effects on ventilation and 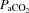 which were similar at rest and during steady-state exercise (Wasserman *et al*. 2001; Forster *et al*. 1981, 1924), the magnitude of further increases in steady-state ventilation induced by exercise does not appear to be dependent upon intact chemoreceptors. There are other promising possibilities. First, the well-controlled experiments of Green *et al*. (Green & Sheldon, 1995) in anaesthetized animals identified pulmonary C-fibres or J receptors in the lung interstitial fluid as a potential site for sensing changes in pulmonary blood flow, with vagally mediated input to the medullary respiratory controller. More recently, Luijendijk (1963) theorized that both pulmonary J receptors and the aortic bodies sensed changes in the osmotic state of plasma, the magnitude of which is determined by pulmonary capillary pressure and plasma osmotic pressure, both of which are indirectly influenced by pulmonary blood flow and excess plasma HCO_3_^−^, and therefore linked to 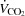. This concept of linking changes in respiratory CO_2_ exchange to J receptor and aortic body stimulation via changes in plasma osmolarity is a promising new twist deserving further study, after all it may provide insight into the mysterious CO_2_ exchange:ventilatory control link which has long been sought.

Is the stimulus driven solely by respiratory CO_2_ exchange obligatory to a normal exercise hyperpnoea or even the major controller of the hyperpnoea? Experimentally this question was addressed by reducing the normal CO_2_ flow to the lung (via an extracorporeal circuit used to scrub the CO_2_ in the venous return) in exercising sheep and dogs (Phillipson *et al*. 1992; Bennett *et al*. 1943). While 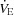 was reduced coincident with a reduced CO_2_ flow, it was controversial among studies to what extent coincident small changes in 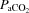 could have accounted for the changes in ventilation.

A fundamental problem with these extremely difficult to conduct studies in awake, exercising animals is that the 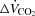 achievable via the extracorporeal circuit was small, thus the accompanying 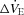 values were in the range that might be explained by small deviations in 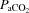 alone (Bennett *et al*. 1943; Dempsey *et al*. 2012). We believe the evidence to date is consistent with the concept that CO_2_ exchange at the lung provides the underpinning for the control of breathing, especially on either side of eupnoea at or near a resting 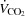 (Menna & Mortola, 1972). However, we know of no evidence that would support a major role for this CO_2_-linked stimulus as the dominant mediator of up to five- to tenfold increases in 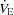 experienced during mild and moderate exercise intensities.

### The ‘work’ factor(s): feedforward and feedback

Erling Asmussen and Marius Nielsen coined this term long ago to suggest that the stimulus to hyperpnoea required a non-humoral component specific to exercise or locomotion *per se* (Asmussen *et al*. 2011b). Two sources have been identified.

First, the ‘central command’ stimulus whereby parallel recruitment occurs of both locomotor muscles and the cardiorespiratory response has been shown to be capable – by itself – of driving the exercise hyperpnoea. Eldridge *et al*. (1985) showed that electrical or pharmacological stimulation of hypothalamic locomotor areas elicited a progressive, robust cardioventilatory response in the decorticate cat, even in the presence of limb musculature under neuromuscular blockade, i.e. ‘fictive locomotion’. Further, in hypnotized humans, at rest, the ‘suggestion’ of increasing exercise intensity prompted immediate hyperventilatory and cardioaccelerator responses and coincident brain-imaging measures showed an increased blood flow to motor control regions of the cortex and cerebellum (Thornton *et al*. 1992; Williamson *et al*. 1980). Direct recordings from electrodes implanted in humans have shown increased neuronal activity in the periaqueductal grey region to accompany the cardiorespiratory response to low level exercise (Green *et al*. 1987). Finally, locomotor muscle weakness achieved via partial curarization elicited hyperventilatory and cardioaccelerator responses to exercise which exceeded those in the intact control subject, presumably via heightened central command mechanisms which are called into play to recruit more motor units and maintain work rate in the face of weakened locomotor muscles (Asmussen *et al*. 2009; Galbo *et al*. 2000). A significant role for central command mechanisms in the normal, intact exercising animal or human has been inferred from studies of this mechanism in isolation. Whether this mechanism is obligatory to the hyperpnoea has not been tested by removing or diminishing this feedforward input during physiological exercise when other potential inputs were operative (also see section below on ‘Exercise hyperpnoea as a learned phenomenon’).

Research on the peripheral ‘feedback’ component of the ‘work’ or locomotor-linked factor in exercise hyperpnoea has provided mixed but mostly negative findings, especially in humans. On the positive side, using anaesthetized canines with cross-circulation preparations, Kao demonstrated a near-normal hyperpnoeic response to limb muscle electrical stimulation, even in the absence of increased CO_2_ flow back to the lung and this ‘neural’ drive effect on 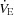 was blocked by denervation of the dorsal spinal columns (Kao, 1989). This mechanism was later shown to be mediated via group III–IV muscle afferents (Coote *et al*. 2009; McCloskey & Mitchell, 2008). Type III–IV muscle afferents were also shown to increase their activity even during low intensity rhythmic contractions (Adreani *et al*. 1984). These afferents project via the dorsal horn of the spinal cord to the nucleus of the solitary tract and the medullary cardiorespiratory controller neurons. Recent evidence in the isolated brain stem–spinal cord rodent preparation confirmed that lumbar locomotor networks can rhythmically entrain medullary respiratory neurons (Morin & Viala, 2002). On the other hand, negative evidence includes the finding of a normal ventilatory response to limb muscle stimulation with increased 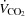 in quadriplegic patients (Adams *et al*. 1984), and spinal cord-transected anaesthetized animals (Weissman *et al*. 2010). In healthy humans, total vascular occlusion of the limbs accelerated the return of ventilation to resting levels during the recovery period following exercise, thereby suggesting that metabolite accumulation in the occluded muscle was not an important contributor to ventilatory drive (Rowell *et al*. 1981b; Innes *et al*. 2011; Haouzi *et al*. 2004a). Several studies have also used epidural lidocaine (lignocaine) in humans to block afferent feedback from the legs during rhythmic exercise (Hornbein *et al*. 1990; Strange *et al*. 2003; Smith *et al*. 1976; Amann *et al*. 2010; Forster *et al*. 1924). This anaesthetic-induced blockade caused a significant reduction in MAP but either no effect or even increases in heart rate or 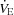 during cycling exercise, suggesting that the muscle afferents were not obligatory to exercise-induced hyperpnoea or cardioacceleration (also see Fig. 6).

## Use of μ-opioid agonists suggests an obligatory contribution of III–IV muscle afferents to steady-state exercise hyperpnoea

Following serendipitous observations from a study concerned with inhibitory feedback effects from fatiguing limb muscles on motor output (Amann *et al*. 2011a) we asked if III–IV muscle afferents were required for a normal cardioventilatory response to rhythmic steady-state exercise in humans. Based on previous demonstrations in anaesthetized animals that μ-opioid agonists blocked much of the cardioventilatory response to static muscle contraction (Hill & Kaufman, 1993) we used lumbar level intrathecal injection of fentanyl (50 μg) in humans to partially block afferent feedback mediated by μ-opioid-sensitive receptors in the dorsal horn. We and others documented with plasma assays, and with ventilatory responses to inhaled CO_2_ and to arm exercise that the fentanyl probably did not spread above the thoracic level or reach the systemic circulation (Amann *et al*. 1984, 2008; Gagnon *et al*. 2012).

**Figure 1:**
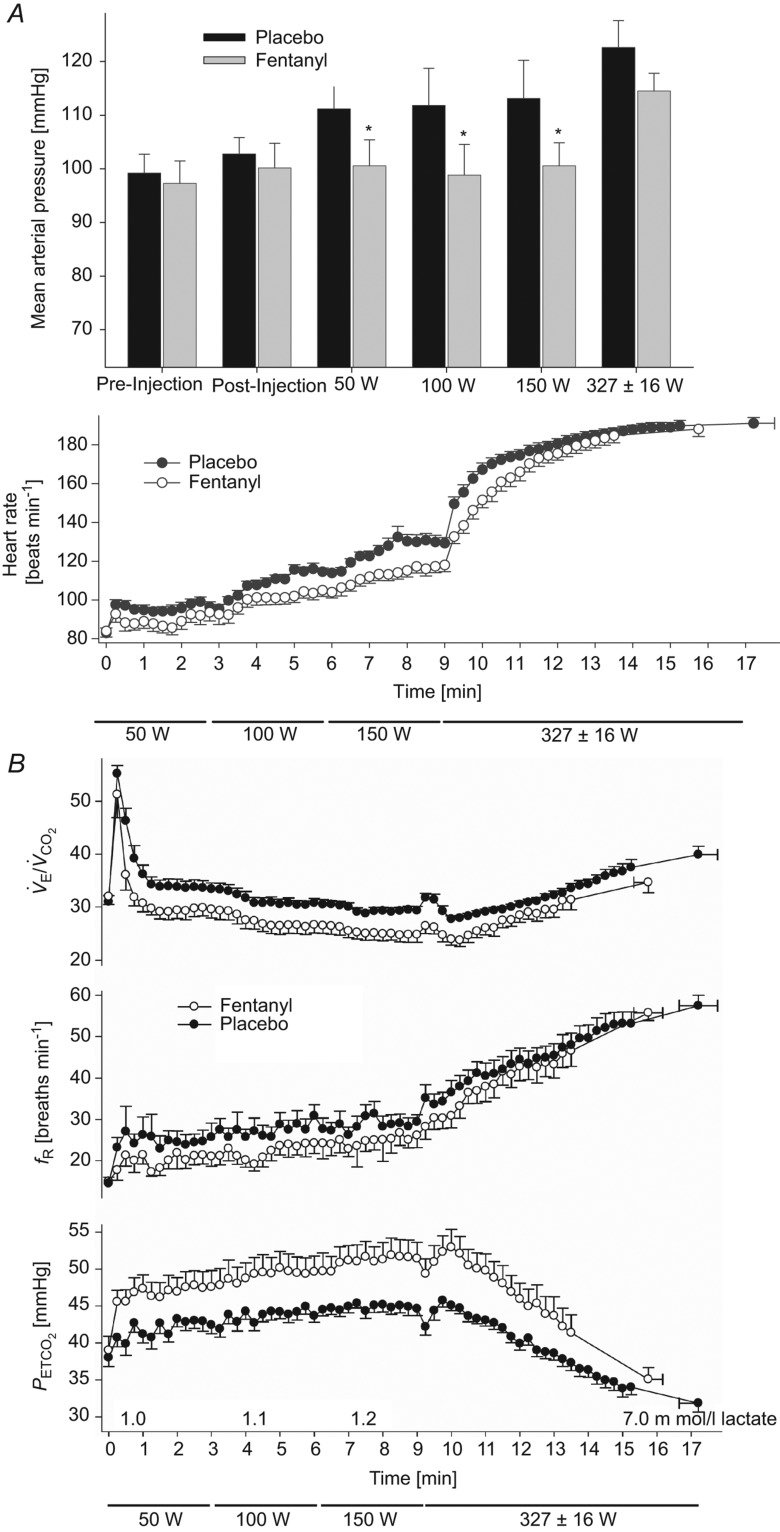
*A*, group mean effects of blockade of type III–IV opiate-sensitive limb muscle afferents on cardioacceleration and mean arterial blood pressure (MAP) at rest and during the transient and steady-state phases of voluntary rhythmic cycling exercise in healthy humans. Data obtained from Amann *et al*. (1984). *B*, reduced steady-state ventilation (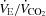 ) and breathing frequency (*f*_R_), and the resultant CO_2_ retention, resulting from type III–IV muscle afferent blockade via intrathecal fentanyl in healthy humans at mild to heavy exercise intensities. Fentanyl had no effect on mean 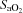 except at the 327 W work rate where 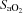 was 97.7% in placebo and 95% with fentanyl. Note the persistence of the hypoventilatory response in the presence of type III–IV afferent blockade – especially during mild and moderate intensity exercise – despite the presence of increased CO_2_-induced chemoreceptor stimulation. Plasma lactate levels were within 0.5 mmol l^−1^ of resting values (0.9 ± 0.1 mmol l^−1^) during 50–150 W exercise and rose to 7-fold > rest during exercise at 325 W in both the placebo and fentanyl trials. Data from Amann *et al*. (1984).

The premise of these studies was to determine the effects of blocking only the type III–IV limb muscle afferent influences during steady-state exercise, i.e. under conditions where all other proposed major stimuli to hyperpnoea (including CO_2_ exchange and central command) would continue unaffected from their normal levels. Based on the time course of changes shown in Fig. 1*A* and *B* it was clear that the heart rate, 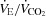, the end-tidal partial pressure of CO_2_ (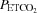 ), and 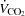 (not shown) achieved a steady state by the final minute of each 3 min session at mild to moderate work intensities, implying that all of the potential stimuli to hyperpnoea were probably operative under these conditions.

**Figure 2:**
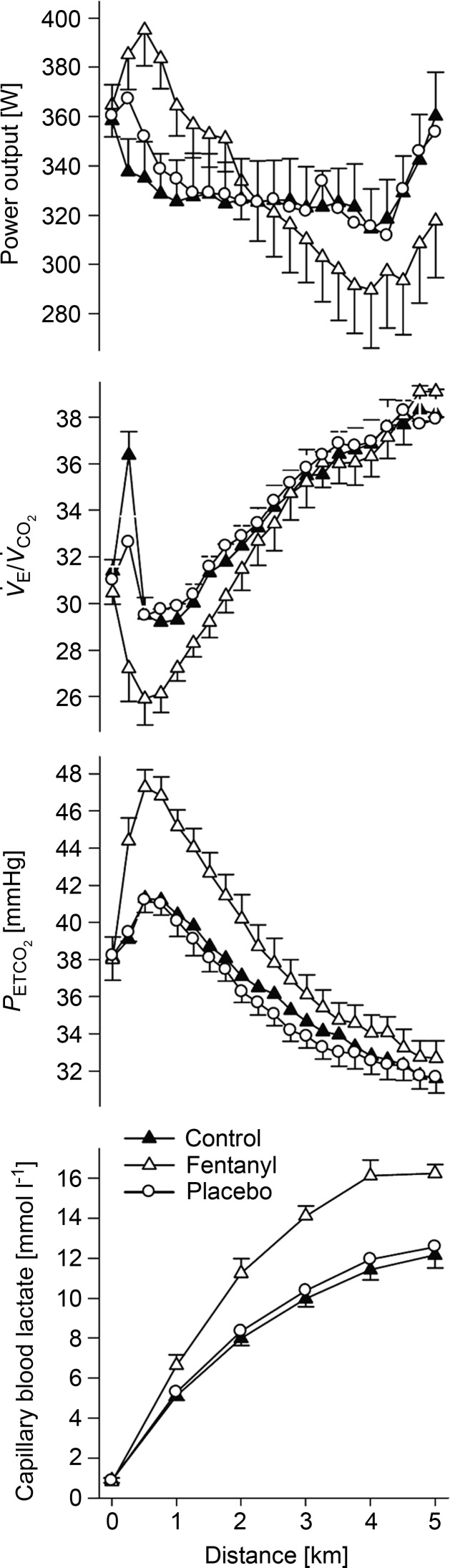
Note the reproducibility in the responses of force output and 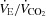 between control and placebo in these trained cyclists throughout the trial. This contrasts with the marked increase in power output (and quadriceps EMG, not shown) ‘chosen’ by the subject over the initial 1–2 km when somatosensory neural feedback was blocked. Thereafter, power output fell as limb fatigue progressed. 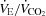 was reduced and 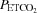 increased throughout the initial 2.5 km in the fentanyl trial *vs*. placebo, but this relative hypoventilation did not persist throughout the trial as plasma lactate rose over the final 2.5 km. 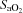 averaged 90–93% over the final 2.5 km *vs*. 95–96% in placebo (data not shown). Data from Amann *et al*. (2011a).

**Figure 3:**
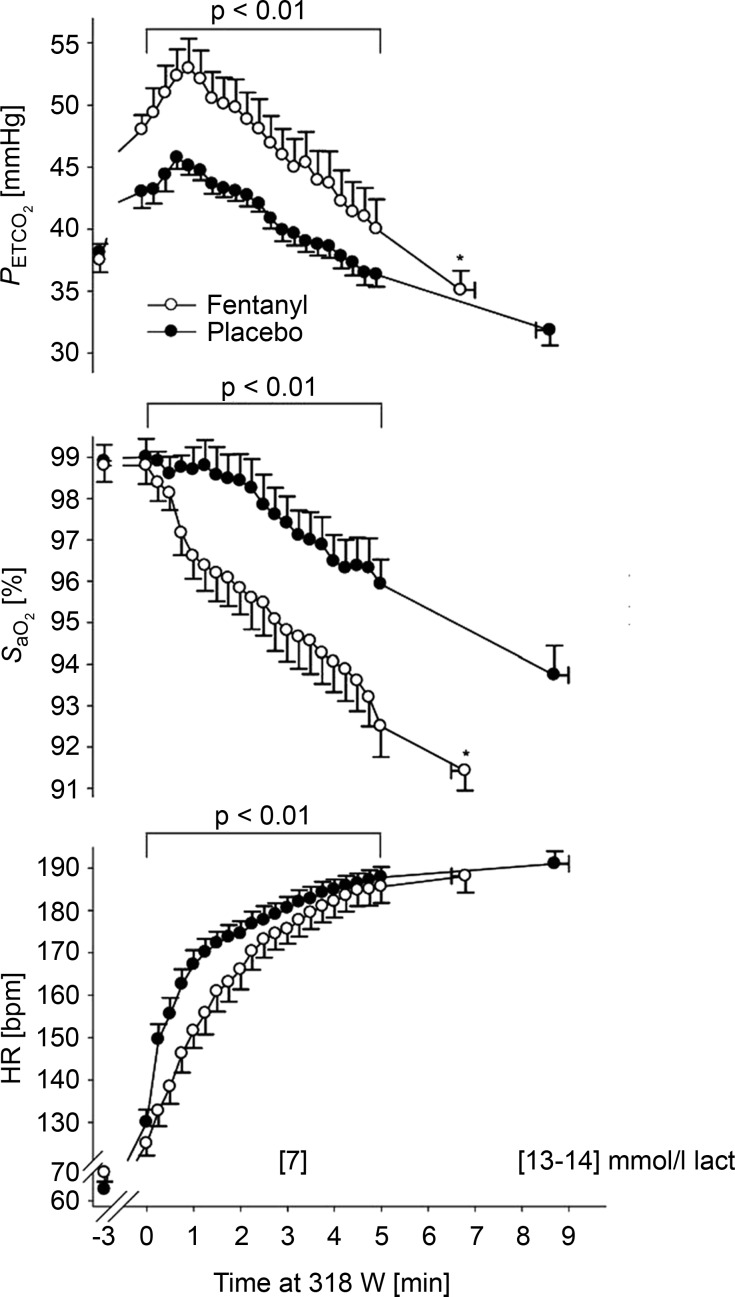
Note the reduced heart rate (HR), along with hypoventilation, CO_2_ retention and arterial hypoxaemia, during the fentanyl trial, which persisted until late in the trial at which time plasma lactate had risen steadily, reaching 13–14 mmol l^−1^ at end-exercise in both placebo and fentanyl trials. Mean arterial blood pressure (not shown) was reduced 7–8 mmHg throughout the fentanyl trial. Data from Amann *et al*. (1997).

**Figure 4:**
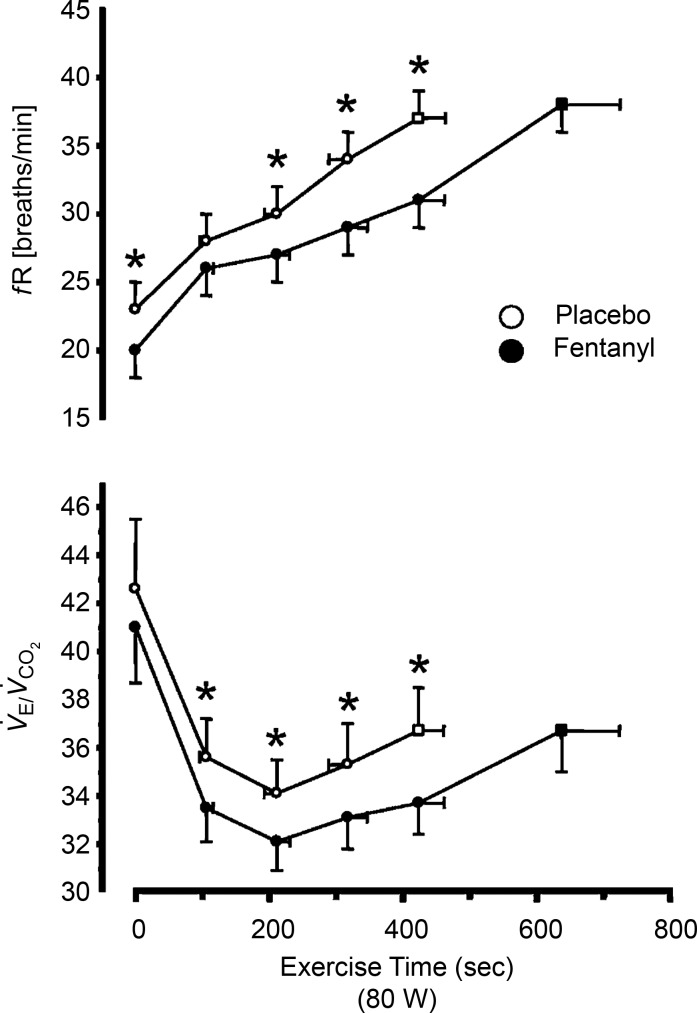
Fentanyl block resulted in a reduced *f*_R_ and 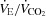 which persisted throughout the exercise. *V*_d_/*V*_T_ during exercise was also reduced with fentanyl (not shown). Dyspnoeic sensations were reduced and exercise time prolonged as 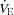 and expiratory flow limitation were reduced with fentanyl blockade. Data from Gagnon *et al*. (2012). Reprinted with permission of the American Thoracic Society. Copyright © 2013 American Thoracic Society.

As shown in Fig. 1*A* and *B* there were no effects of the fentanyl observed at rest but throughout each of the three mild to moderate intensity cycling exercise levels, mean arterial blood pressure (MAP), heart rate, breathing frequency (*f*_R_) and 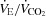 were significantly reduced below placebo levels and 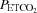 substantially elevated. This sustained hypoventilation and the CO_2_ retention resulting from type III–IV afferent blockade with fentanyl were also obtained during the first half of a 5 km time trial cycling exercise (Amann *et al*. 2011a; see Fig. 2) and over most of the duration of constant load heavy intensity cycling exercise (see Fig. 3; Amann *et al*. 1997). Similarly, use of the intrathecal fentanyl block in chronic obstructive pulmonary disease (COPD) patients caused *f*_R_, 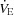, heart rate and MAP to be substantially reduced throughout the entire duration of a constant-load high intensity cycling exercise (see Fig. 4). In these patients *V*_d_/*V*_T_ during exercise with the fentanyl block was also reduced, thus only minimal CO_2_ retention occurred during exercise with afferent blockade (Gagnon *et al*. 2012).

## The ‘relative’ influence of type III–IV muscle afferent feedback with changing exercise intensities/durations/muscle mass

During mild to moderate exercise intensities with type III–IV afferent blockade we observed a 3–10 l min^−1^ reduction in 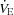 and 3–8 mmHg increases in 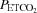 (*vs*. placebo), as well as significant reductions in heart rate and the prevention of any increase in MAP above resting levels. These effects were present within the initial 15 s of each work rate and persisted throughout the ensuing 3 min of each load and for a cumulative 12–15 min of exercise over several loads (see Fig. 1*A* and *B*). The sustained hypoventilation and accompanying hypercapnia (and reduced arterial oxygen saturation, 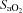 ) with type III–IV afferent blockade does not mean that chemoreceptors remained non-responsive to this substantial systemic (and brain) acidity. Rather, even greater reductions in 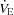 and heart rate with type III–IV afferent blockade were probably masked by several secondary factors, including: (a) the concomitant and substantial chemostimulation secondary to increasing 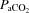 (and reduced arterial O_2_ saturation), which undoubtedly prevented 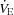 from falling further secondary to the fentanyl blockade (Bennett & Fordyce, 1965); and (b) a lower MAP and therefore unloading of the carotid sinus and aortic baroreceptors which via feedback effects will increase both heart rate and the drive to breathe (Ohtake & Jennings, 2006).

**Figure 5:**
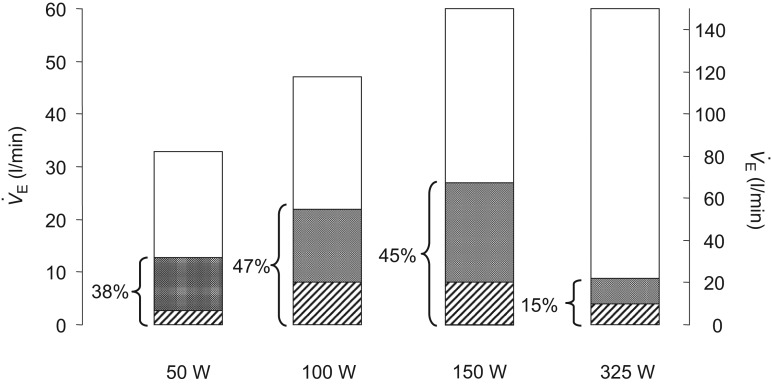
The left-hand *Y*-axis pertains to 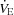 during the final minute at 50, 100 and 150 W exercise and the right-hand Y-axis pertains to 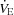 during 325 W exercise. The total height of each column denotes the total ventilatory response to each of the exercise loads. The lowest, hatched area of each bar is the 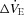 observed with fentanyl *vs*. placebo; the dark area represents the estimated 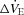 due to the CO_2_ retention obtained with fentanyl block during exercise (as determined by the subjects’ ventilatory response to increased inspired CO_2_ fraction measured at rest, i.e. 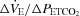 = 2.4 ± 0.5 l min^−1^ mmHg^−1^); the open areas represent the remaining 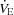 not affected by the fentanyl blockade and the resultant CO_2_ retention. As indicated, the hyperpnoea attributable to type III–IV muscle afferent input averaged 38–47% at mild and moderate exercise intensities and ∼15% in heavy intensity exercise (also see text). Data from Amann *et al*. (1984) and as shown here in Fig. 1.

In Fig. 5 we calculate the relative contributions of type III–IV muscle afferents to the total hyperpnoea by including estimates of the ‘masking’ effect of one of these feedback influences, i.e. the coincident hypercapnia, on the hyperpnoeic response. For this estimate we used the group mean ventilatory response to inhaled CO_2_ measured at rest (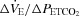; Amann *et al*. 1984). Note that an average of 38–47% of the total steady-state ventilatory response to mild to moderate intensity cycling exercise was attributed to the type III–IV muscle afferent input. Given that we only accounted for one of the secondary feedback effects (i.e. hypercapnia) and that we have only blocked the μ-opiate-sensitive afferents, this estimate probably represents a minimum effect of muscle afferent feedback effects on the hyperpnoea.

As exercise intensity increased and/or exercise duration at high intensity was prolonged, the relative effects of afferent blockade on the hyperventilatory, cardioaccelerator and MAP responses were clearly diminished. We attribute this apparent reduction in the contribution of III–IV afferents to two factors. First, these reduced effects of opiate agonists on 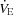 with increasing exercise intensities/durations did not mean that type III–IV muscle afferents were decreasing their absolute activity. On the contrary, just the opposite effect would be expected with accumulation of muscle metabolites. Thus, it is more likely that our partial blockade of opiate-sensitive type III–IV afferents became progressively more incapable of suppressing hyperpnoea in the face of rising muscle metaboreceptor stimuli. Secondly, based on the observed increase in plasma lactate (see Figs 1*B*, 2 and 3) we speculate that this reduced relative influence on 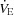 from III–IV afferents was due in part to the addition of two types of overriding influences, namely: (a) increased chemoreceptor stimuli such as circulating H^+^, K^+^ and noradrenaline (Paterson *et al*. 2002; Forster *et al*. 1924); and (b) the augmented central command influences which would have accompanied attempts to maintain force output as muscle fatigue progressed. Given these additional causes of hyperventilation during heavier intensity exercise and the increasing uncertainty of the relative adequacy of our type III–IV muscle afferent blockade in the face of rising muscle metaboreceptor stimuli, we are unable to attribute the calculated 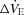 in Fig. 5 at the heaviest intensity solely to the effects of muscle afferent feedback and its sequelae.

Finally, we note that fentanyl blockade during exercise using a single leg kick (Amann *et al*. 2008) elicited a significant although relatively small hypoventilatory effect, perhaps revealing the important influence of muscle mass on the role of muscle afferents in hyperpnoea (Rowell & O'Leary, 2007). These data, as well as those obtained during high intensity prolonged exercise (Amann *et al*. 2008), also revealed a relatively greater effect of III–IV afferent blockade on reducing the arterial pressure response to exercise (probably via a reduced sympathetic vasoconstrictor response) than on reducing the exercise hyperpnoea.

## Why do the fentanyl effects on hyperpnoea differ from previous negative findings concerning contributions from muscle afferents to exercise hyperpnoea?

**Figure 6:**
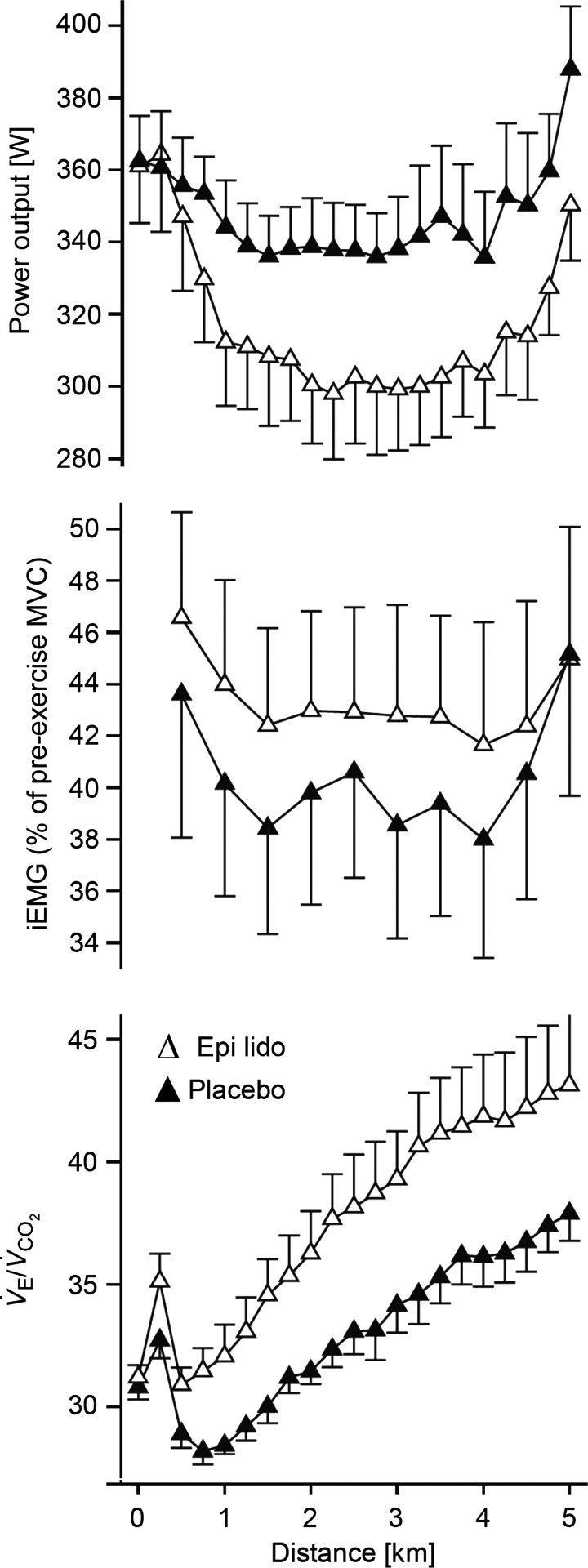
With epidural lidocaine, prior to cycle exercise, maximum voluntary contraction (MVC) force output was reduced 22% and percentage voluntary activation reduced from 97 to 81%. Throughout the time trial with epidural lidocaine, power output was reduced but quadriceps EMG (iEMG) and 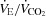 were elevated, as was heart rate at any given 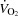 (not shown). Data from Amann *et al*. (2010).

We propose that the recent fentanyl blockade findings in health and COPD reveal an obligatory role in steady-state exercise hyperpnoea for group III–IV muscle afferent feedback. These data and interpretations differ from previous negative findings (see ‘The work factor’ section above) for the following reasons. First, epidural anaesthetics (see Fig. 6) block efferent (ventral horn) as well as afferent (dorsal horn) neurons resulting in a 20–40% decrement in strength of the lower limbs. Accordingly (as with the effect of curare (Asmussen *et al*. 2009; Galbo *et al*. 2000), to maintain a given power output, central command is augmented in order to recruit more motor units. The associated parallel increases in respiratory motor output (i.e. corollary discharge) would result in no (net) effect of afferent blockade or even a greater hyperventilatory and/or heart rate response (see Fig. 6; Smith *et al*. 1976; Amann *et al*. 2010). On the other hand intrathecal fentanyl blocks only muscle afferents and leaves leg strength unaffected (Amann *et al*. 2011a, 1984, 1997; Hilty *et al*. 2006). Presumably then, this μ-opiate receptor agonist partially blocks afferents from a contracting muscle while leaving the remaining primary feedforward stimuli to hyperpnoea unchanged.

Secondly, reports of an accelerated decline of ventilation during recovery from exercise in the face of total vascular occlusion may be explained by two limitations of experimental design. First, there are important interactive effects between mechano- and metabosensitive afferents in a contracting muscle (Hayes *et al*. 2004b), which would not be present in a muscle at rest, i.e. during recovery from exercise. Secondly, the use of *total* vascular occlusion may have actually reduced (rather than augmented) type III–IV afferent activity in contracting limb muscles as demonstrated by the reduction in 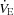 accompanying arterial occlusion alone *vs*. the hyperpnoea which accompanied venous occlusion alone (Haouzi *et al*. 2004a, 1983) (also see ‘Mechanisms underlying afferent contributions’ section below regarding the effects of venous distension).

It is encouraging that significant effects of intrathecal fentanyl on the cardiorespiratory response occurred with different types of whole body exercise in health (see Figs 3) and even in disease states such as COPD (Fig. 4). On the other hand many unknowns remain. Importantly, we do not know to what degree the total III–IV afferent feedback was blocked in any of these experiments – presumably only those, or a portion of those, sensitive to μ-opioid agonists (Hill & Kaufman, 1993). Nor do we know whether the substantial cardiorespiratory effects we have observed are secondary to blockade of the supraspinal pathway from the dorsal horn to the nucleus of the solitary tract and medullary rhythm-generating neurons and/or whether we have interfered with the interactive effects of ascending afferents on descending ‘central command’ influences at the level of the brain stem or spinal motor neurons (Garland & Kaufman, 2012; Dempsey, 1971).

We recognize that there is currently only a very limited amount of data testing the role of group III–IV afferents in cardiorespiratory control in the exercising human using methods which avoid secondary effects on central command mechanisms. Further studies need to consider drug dosages, alternative methods of blockade, means of assessing the degree of total (afferent) blockade and the use of exercise of varying durations, intensities and muscle mass. Also, given the substantial effects of type III–IV muscle afferent blockade in COPD patients on the cardioventilatory response as well as on central locomotor output during exercise (Gagnon *et al*. 2012; see Fig. 4), similar protocols need to be applied in other chronic diseases such as congestive heart failure (CHF). In animal models of CHF, an enhanced sensitivity of muscle mechanoreceptors to muscle contraction has been reported and implicated in their sensitized sympathetic response to exercise (Wang *et al*. 1993).

## Mechanisms underlying muscle afferent contributions to the cardiorespiratory response to rhythmic exercise

The cardiorespiratory effects of fentanyl blockade occurred during rhythmic exercise with normal muscle blood flow under steady-state exercise conditions and at intensities where O_2_ supply met O_2_ demand. Two means of recruiting muscle metaboreceptors might occur under these conditions. First, group III–IV afferents are activated via accumulation of multiple muscle metabolites, such as protons, ATP and lactate – even during moderate-intensity rhythmic exercise (Light *et al*. 1991). Secondly, as suggested by Haouzi *et al*. (2004a, 2005) and the recent work of Cui *et al*. in humans (Cui *et al*. 1978), venous distension – which in turn would change in proportion to muscle blood flow – might also be a key mediator of increasing muscle metaboreceptor activity during rhythmic exercise even of mild to moderate intensity (Haouzi *et al*. 2005). This mechanism is also an appealing one teleologically, because the proposed stimulus, i.e. blood flow, is a major determinant of CO_2_ flow and respiratory CO_2_ exchange and is, therefore, participating (in part) in its own control. According to Haouzi *et al*. such a regulatory system ‘…anticipates the chemical changes that occur in the arterial blood during increased metabolic load and attempts to minimize them by adjusting 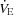 to muscle perfusion, thus matching the magnitudes of the peripheral and pulmonary gas exchange’ (Haouzi & Chenuel, 2007; Forster *et al*. 1924). This proposal needs in-depth study with an approach capable of quantifying the magnitude of cardioventilatory effects of these proposed muscle metaboreceptor stimuli in conditions approximating physiological exercise.

## Exercise hyperpnoea as a ‘learned’ phenomenon?

The afferent blockade findings have a bearing on the concept of central command as a ‘learned’ phenomenon pertaining to both the drive to locomotor muscles and to breathing. This idea has emerged, at least in part, because of the failure to demonstrate ‘obligatory’ reflex contributions to the exercise hyperpnoea (Yamamoto, 2002). For example, Somjen (1990) reasoned that the medullary respiratory neurons are incapable of solving the complex differential equations associated with simultaneous input from multiple peripheral reflexes; thus the higher CNS must rely on stored information learned from past experiences and mistakes to ‘anticipate present and future needs’. Thornton *et al*. also suggested, from their identification in humans of cortical areas involved in central command (see above), that an ‘error free’ ventilatory response to exercise must be accompanied by ‘adaptive feedforward control’ (Thornton *et al*. 1992). Further, Poon *et al*. postulated an ‘emergent controller signal encoding the projected metabolic requirement’, which incorporates an ‘internal model, self-tuning adaptive control paradigm’ (Poon *et al*. 1981a). Experimentally, the idea of memory-like contributions to exercise hyperpnoea is supported by the ‘associative learning’ induced via serotonin-dependent mechanisms underlying long-term modulation of the ventilatory response to exercise (Mitchell & Babb, 2012).

These well-argued, exciting and plausible concepts are certainly worth exploring as knowledge of the higher CNS evolves. However, we interpret the effects of muscle afferent blockade summarized here to mean that this feedforward control system – even in the ‘experienced’ CNS – cannot provide anything even close to error-free control *by itself*! The best example to support this point may be found in the time-trial cycling experiments wherein subjects had only the goal of performance time and were free to ‘choose’ their pattern of power output over time. Over several repeat trials on different days (i.e. see control and placebo examples in Fig. 2), these highly experienced competitive cyclists ‘chose’ almost identical, temporal patterns of power output, and experienced near-identical patterns of cardioventilatory responses and limb fatigue development over the 5 km distance. However, in the absence of type III–IV afferent feedback, these subjects: (a) greatly overshot their normally ‘preferred’ central motor output to the locomotor muscles, thereby causing premature and excessive locomotor muscle fatigue; (b) hypoventilated with accompanying hypercapnia and hypoxaemia; and (c) as shown in additional studies (Amann *et al*. 1997,2008), significantly reduced limb blood flow and perfusion pressure, and O_2_ transport to working muscle. Thus, it is not unreasonable to believe that ‘experience’ must have provided significant, anticipatory, feed-forward forms of guidance in these athletes. On the other hand, these data point to afferent feedback as a key determinant of central control over locomotor muscle performance as well as key cardiorespiratory responses, even in ‘well-rehearsed’ performers.

## Hypothesis

Although several mechanisms which are present during exercise have been shown to drive ventilation when each is studied in isolation, they have not shown an obligatory contribution to the normal hyperpnoea when manipulated in the presence of other ‘competing’ stimuli during physiological exercise (Yamamoto, 2002). In order to determine whether type III–IV muscle afferents were obligatory to steady-state exercise hyperpnoea we proposed that this proof required: (a) a setting of physiological steady-state exercise, i.e. during which all potential stimuli are operative; and (b) a reduction in the hyperpnoeic response upon removal/reduction of the stimulus in question without significant alteration of coexisting inputs. We believe the findings employing an intrathecal opiate agonist in several different types of exercise and subjects to date support the hypothesis of an essential contribution of type III–IV muscle afferents to the normal hyperpnoea experienced during mild to moderate rhythmic, large muscle exercise in the human. These findings do not rule out major contributions to the hyperpnoea from other proposed stimuli. Indeed when studied in isolation, CO_2_ respiratory exchange and central command elicit significant, precise influences on ventilatory control (see section ‘Three key mechanisms’ above). It remains to be determined what role respiratory CO_2_ exchange-related stimuli and/or central command might play as modulators and/or primary drivers of the hyperpnoea when they coexist with each other and with muscle afferent influences during physiological exercise. In anticipation, we would predict a significant interactive effect on the cardioventilatory response to occur between muscle afferent feedback and feedforward central command, with a strong secondary modulatory ‘fine-tuning’ effect of a mechanism specific to CO_2_ exchange sensed at the lung. As judged by the substantial ‘feedback’ we have received from many investigators since our first opiate agonist findings were published, it is abundantly clear that many fundamental questions remain. Our hope is that innovative research at the molecular to integrative levels will experience a much needed rejuvenation on this hyperpnoea problem that is so very fundamental to the field of cardiorespiratory control.

## Notes

**Jerome A. Dempsey, PhD.** was born and educated in Canada (Universities of Western Ontario and Alberta) and received his PhD at the University of Wisconsin-Madison. He has been on the faculty at UW Medical School since 1968, director of the John Rankin Laboratory of Pulmonary Medicine since 1981 and is currently Professor Emeritus. His major interests have included the respiratory physiology, pathophysiology and cardiorespiratory interactions attending exercise, hypoxia, sleep, obesity, COPD and CHF and the training of pre- and post-doctoral fellows and undergraduates.
